# Dual-neodymium magnet-based microfluidic separation device

**DOI:** 10.1038/s41598-019-45929-y

**Published:** 2019-07-01

**Authors:** Hyeon Gi Kye, Byeong Seon Park, Jong Min Lee, Min Gyu Song, Han Gyeol Song, Christian D. Ahrberg, Bong Geun Chung

**Affiliations:** Department of Mechanical Engineering, Sogang University, Seoul, 04107 Republic of Korea

**Keywords:** Lab-on-a-chip, Biomedical engineering

## Abstract

Microfluidic-based separation methods have been highlighted for a number of biological applications, such as single cell analysis, disease diagnostics, and therapeutics. Although a number of previous studies have been carried out to minimize the physical damage and chemical deformations of the sample during the separation process, it still remains a challenge. In this paper, we developed a microfluidic device with dual-neodymium magnet-based negative magnetophoresis for the separation of the microparticles and cells. The poly(ethylene oxide) (PEO) was added to the solution to increase the viscoelasticity of the medium which could assist the sorting of the microparticles in the microfluidic device even at low flow rates, while minimizing damage to the cells and microparticles. Following this method, it was possible to separate 10 and 16 μm microparticles with high efficiency of 99 ± 0.1%, and 97 ± 0.8%, respectively. We also demonstrated the separation of glioblastoma cancer cells and neural stem cells (NSCs) in the microfluidic device.

## Introduction

Microfluidic devices have been widely used for various biomedical applications due to their unique advantages, such as fast detection, easy operation, and low cost^1–5^. In particular, the ability of microfluidic devices to isolate individual cells or particles for further analysis has been of great interest for single cell analysis applications^6,7^. The separation of microparticles in microfluidic devices has previously been reported by following methods: fluidic flow^8–11^, antibody^12,13^, surface acoustic wave (SAW)^14–17^, and magnetic force^18,19^. When the fluidic flow inside the microfluidic devices is used for the separation of microparticles or cells, this can be done without the requirement of applying an external force. However, for the size-dependent separation inside these microfluidic devices, high flow rates have to be used. It can lead to physical damage of the particles or cells. Furthermore, the separation of circulating tumor cells (CTCs) or cell free DNA (cfDNA) from blood samples at high flow rates can cause hemolysis, which negatively influences analysis and detection. To prevent physical damage to the cells from the shear flow, antibody-based separation methods have been developed. Through these methods, it is possible to specifically separate the target cells due to the selective reaction of the antibody. However, it requires a complicated antibody reaction process and is also difficult to separate various cell types simultaneously using a single separation step. To overcome these limitations, the separation of microparticles using SAW method was developed. By introducing SAW to microfluidic devices, it is possible to separate and capture the samples in a highly efficient manner. Also, the SAW methods enable separation of target biomarkers without any dilution and blood culture steps^17^. Furthermore, the real-time analysis and feedback systems can be constructed by using a controller-modulating SAW. However, these SAW-based microfluidic systems are sensitive to environmental factors, such as vibrations or noises.

The magnet-mediated separation of the microparticles or cells in microfluidic devices has recently been developed by following two approaches: separating magnetic microparticles from non-magnetic microparticles or enriching the magnetic microparticles using magnetic field (positive magnetophoresis)^18,20–22^ and size-based separation of non-magnetic microparticles suspended in a ferrofluid (negative magnetophoresis)^19,23–25^. Negative magnetophoresis imposes no limitations on the size or shape of the microparticles or cells. Therefore, the biocompatible ferrofluid-based system can be used for cell separation in microfluidic devices. Through a force provided by an external magnet, the microparticles suspended in the ferrofluid are sorted according to size within the microchannel by the negative magnetophoretic force^26^. Although negative magnetophoresis-based separations have been demonstrated for the separation of microparticles and cells, high cell densities cause a significant drop in separation efficiency. This limitation could be addressed by repeating the separation process in the microfluidic device, adjusting the separation threshold with each repetition. However, the concentration of the ferrofluid is decreased after each pass of the magnetic-based microfluidic sorting device. Furthermore, the use of two or more magnets in a microfluidic device could lead to an inhomogeneous magnetic field which could affect the position of the microparticles in the microchannels. Alternatively, the equilibrium between a viscoelastic force and the internal lift force of the particles can be used for aligning the particles in the microfluidic device. In practice, this is achieved through non-Newtonian flow obtained through the addition of long-chain polymers to the solution containing the particles^27–29^. For example, microparticles can be aligned to the center or edge of the microchannel by increasing the viscosity of an aqueous solution using poly(ethylene oxide) (PEO) or poly(vinyl pyrrolidone) (PVP)^27,30–32^. However, even when using a combination of negative magnetophoresis and non-Newtonian viscoelastic flow, there are some remaining challenges to separate samples with high cell densities^33–35^. In this case, the interference of the flow by cell-cell interactions can no longer be ignored. To overcome this problem, a dilution step is required prior to sample separation. However, the dilution step can reduce the sensitivity of the microfluidic separation device. In addition, the large sample volume from the dilution step also requires longer separation times and larger volume fluid reservoirs. Thus, the development of the simple separation method without any pre-dilution step in the microfluidic device is required.

In this paper, we developed a microfluidic device with dual-neodymium magnet-based negative magnetophoresis for the separation of microparticles and cells. Through the addition of a sorting region, we decreased the cell density in the total sample while increasing the cell density of the target cells without requiring any pre-dilution step. The microparticles in the medium passing through the sorting step were completely separated in the consecutive separation step. As the separation process is repeated, the sensitivity of the microfluidic device can be improved and the stepwise separation can minimize the effects caused by high concentration of the microparticles and cells. We further added PEO solution to increase viscoelasticity of the medium which could assist the sorting of the microparticles in the microfluidic device even at low flow rates. Therefore, this microfluidic device combines the advantages of both viscoelasticity-based and negative magnetophoresis methods.

## Mechanisms

### Negative magnetophoretic force

If the non-magnetic particles suspended in a ferrofluid enter an inhomogeneous magnetic field, they experience a negative magnetophoretic force. This force can be described by the following equation:

1 $${F}_{m}=3\mu {}_{0}V_{p}\frac{{\chi }_{p}-{\chi }_{f}}{3+{\chi }_{p}+{\chi }_{f}}(H\cdot \nabla )H$$

where *μ*_0_ = 4*π* × 10^−7^ Hm^−1^ presents the permeability of free, *V*_*p*_ is the volume of the microparticle, *χ*_*p*_ is the magnetic susceptibility of microparticle, *χ*_*f*_ is the magnetic susceptibility of ferrofluid, and *H* is the magnetic field at the center of the microparticle. As the microparticles are non-magnetic, the magnetic susceptibility of the microparticles is smaller than that of the ferrofluid, *χ*_*p*_ < *χ*_*f*_. The direction of magnetophoretic force *F*_*m*_ is along the inverse of the gradient of magnetic field. When the magnitude of *χ*_*p*_ and *χ*_*f*_ are much smaller than 1, the equation can be simplified as equation^36^:

2 $${F}_{m}={\mu }_{0}{V}_{p}({\chi }_{p}-{\chi }_{f})(H\cdot \nabla )H$$

### Inertia lift force

Particle trajectories in Newtonian flow are governed by two factors: the shear-induced force and the wall-induced force. The equilibrium of these two forces governs the alignment of the particles inside the microchannel. This equilibrium can be expressed by the net inertial lift force, which can be calculated by^37,38^:

3 $${F}_{L}=\frac{{\rho }_{f}{U}_{m}^{2}{a}^{4}}{{D}_{h}^{2}}{f}_{L}({R}_{c},{x}_{c})$$

4 $${R}_{c}=\frac{{\rho }_{f}{U}_{m}{D}_{h}}{{\mu }_{f}}=\frac{2{\rho }_{f}Q}{{\mu }_{f}(w+h)}$$

Where *ρ*_*f*_, *U*_*m*_, and *μ*_*f*_ are density, mean velocity, and dynamic viscosity of the fluid, respectively. *a* is the diameter of the spherical microparticles and *D*_*h*_ the hydraulic diameter. For the rectangular channels, it can be expressed as ${D}_{h}=\frac{2wh}{(w+h)}$, where *w* is the width and *h* is the height of the microfluidic channel. The lift coefficient *f*_*L*_(*R*_*c*_, *x*_*c*_) is a function of the position of the microparticle within the cross-section of the microchannel *x*_*c*_ and the channel Reynolds number *R*_*c*_^38^. *Q* represents the liquid flow rate.

### Viscoelastic force

In case of non-Newtonian flow, an additional force is acting upon the microparticles, so-called viscoelastic force. For this purpose, the Weissenberg number Wi was introduced. It is the ratio of elastic to viscous forces. It can be calculated through the ratio of relaxation time and the characteristic time of the microchannel as follows^39^:

5 $${\rm{Wi}}=\frac{\lambda }{{t}_{r}}=\lambda \dot{\gamma }=\lambda \frac{2{U}_{m}}{w}=\frac{2\lambda Q}{h{w}^{2}}$$

Where *λ* is the relaxation time, *t*_*r*_ is the characteristic time of microfluidic channels, and $\dot{\gamma }$ is the average shear stress of the fluid. The characteristic time of the microfluidic channel is equal to inverse of the average shear stress, which can be expressed as $\frac{2Q}{hw}$ in a rectangular channel. The relaxation time of 2 × 10^6^ Da PEO solution is experimentally obtained using the droplet formation and breakup method within a time scale on the order of 1.78 to 2.82 ms. Experimentally determined values were similar to previous studies^40,41^. In a viscoelastic fluid, both of the first and second normal stresses, *N*_1_ = *τ*_*xx*_ − *τ*_*yy*_ and *N*_2_ = *τ*_*yy*_ − *τ*_*zz*_, contribute to particle migration, where *τ*_*xx*_, *τ*_*yy*_, and *τ*_*zz*_ are the normal stress in the flow direction. Because *N*_1_ is much larger than *N*_2_ in the diluted PEO solution, the effects of *N*_2_ can be neglected. The viscoelastic force *F*_*E*_ originates from an imbalance in the distribution of *N*_1_ over the volume of the particle^39,42,43^.

6 $${F}_{E}\sim {a}^{3}\nabla {N}_{1}={a}^{3}(\nabla {\tau }_{xx}-\nabla {\tau }_{yy})$$

In case of viscoelastic flow, Wi and Re increase with increasing flow rate. The microparticle trajectory is determined by the relative weight of viscoelasticity and inertia. It can be characterized by the elasticity number *El* which is expressed as:

7 $$El=\frac{Wi}{{\rm{Re}}}=\frac{\lambda \mu (w+h)}{\rho {w}^{2}h}$$

If *El* is approaching 0, the inertial lift force is dominant. As *El* is significantly larger than 1, we can assume the inertial lift force as negligible and only consider the viscoelastic force^39^.

### Hydrodynamic drag force

Due to the small scale of microfluidic systems liquid flow inside of these channels can typically be considered as laminar with low Reynolds numbers. The hydrodynamic drag force, originating from the velocity difference between the fluid and microparticles, played a dominant role in determining the microparticle and cell movement. The hydrodynamic drag force can be expressed by the following equation:

8 $${F}_{D}=3\pi {\mu }_{f}a({v}_{f}-{v}_{p}){f}_{D}\sim 3\pi {\mu }_{f}a({v}_{f}-{v}_{p})$$

Where *v*_*f*_ and *v*_*p*_ are the velocities of the fluid and microparticle, respectively. *f*_*D*_ is the hydrodynamic drag force coefficient which is a function of particle diameter and distance to the closest channel wall. Under low ferrofluid concentrations, this factor can be considered as 1, as the magnetoviscous effect can be neglected under these cirumstances^44–48^.

### Microparticle dynamics

The microparticles suspended in a viscoelastic ferrofluid experience a negative magnetophoretic force as well as inertia lift force, viscoelastic force, and hydrodynamic drag force. The sum of those forces results in an acceleration of the particles according to the following equation:

9 $${m}_{p}\frac{d{v}_{p}}{dt}={F}_{m}+{F}_{L}+{F}_{E}+{F}_{D}$$

Where *m*_*p*_ is the mass of microparticles. As we are dealing with a microfluidic system the effect of gravity, the buoyancy is small compared to the other forces and can be neglected. In our microfluidic design, the viscoelastic force *F*_*E*_ and inertial lift force *F*_*L*_ are used for sorting of microparticles and the magnetophoretic force *F*_*m*_ is used for size-based separation of microparticles. To characterize the particle motion, we introduce to dimensionless numbers. The first dimensionless number Π_*m*_ represents the ratio of negative magnetophoretic force *F*_*m*_ to inertial lift force *F*_*L*_. The second dimensionless number Π_*E*_ represents the ratio of viscoelastic force *F*_*E*_ to inertial lift force *F*_*L*_. The two dimensionless numbers are calculated by:

10 $${\prod }_{m}=\frac{{F}_{m}}{{F}_{L}}=\frac{{\mu }_{0}{V}_{P}{{D}_{h}}^{2}({\chi }_{p}-{\chi }_{f})(H\cdot \nabla )H}{{\rho }_{f}{{U}_{m}}^{2}{a}^{4}{f}_{L}({R}_{c},x{}_{c})}$$

11 $${\prod }_{E}=\frac{{F}_{E}}{{F}_{L}}=\frac{{{D}_{h}}^{2}(\nabla {\tau }_{xx}-\nabla {\tau }_{yy})}{{\rho }_{f}{{U}_{m}}^{2}{a}^{3}{f}_{L}({R}_{c},x{}_{c})}$$

When the flow velocity is increased, Π_*m*_ and Π_*E*_ are decreased, if all other parameters remain constant.

## Material and Methods

### Fabrication of the microfluidic separation device with dual-neodymium magnets

**Figure 1:**
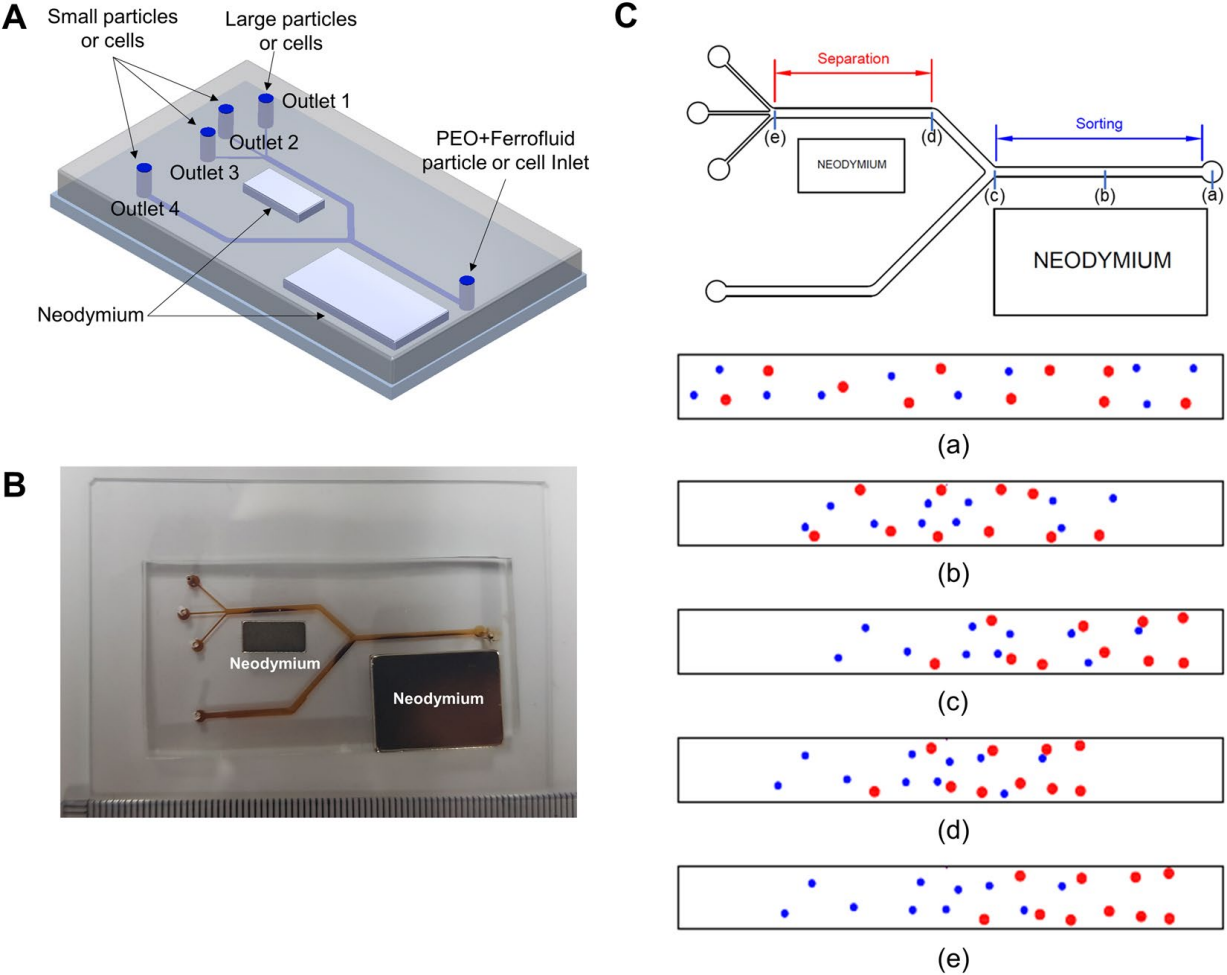
Schematic drawing of the microfluidic separation device. (**A**) 3D CAD image of the microfluidic device for size-dependent separation of the microparticles and cells. (**B**) Photograph of the microfluidic separation device with dual-neodymium magnets. (**C**) Schematic drawing of the microparticle distribution within the microfluidic channel. The red dots represent the larger size microparticles and blue dots represent the smaller size microparticles.

The microfluidic separation device was designed using Autocad (Autodesk, USA) and was subsequently fabricated by the soft lithography process using negative photoresist SU8-100 (Microchem Corp., USA) with a height of 100 µm. The microfluidic channel consisted of a sorting and a separation region with dimension of 900 µm × 20 mm and 900 µm × 15 mm, respectively. Dual-neodymium magnets with different sizes were used, the first neodymium magnet (20 mm × 10 mm size, 2000 Gauss, JSMAGNET, Korea) for the sorting process was placed parallel to the sorting region with a lateral distance of approximately 3 mm. The second neodymium magnet (10 mm × 5 mm size, 2000 Gauss, JSMAGNET, Korea) for separation process was placed on the same side as the first neodymium, parallel to the separation region. The three-dimensional (3D) illustration image of the microfluidic device and the image of fabricated microfluidic device are shown in Fig. 1.

### Preparation of ferrofluid-based viscoelastic solution

PEO (MA 2,000,000 Da, Sigma-Aldrich, Australia) was dissolved in phosphate buffered saline (PBS, Thermo Fisher Scientific, USA) to sort the microparticles in the microfluidic channel. Tween 20 (0.01% (v/v), Sigma-Aldrich, Australia) was added to the prepared PEO solution to prevent the aggregation of the microparticles. To generate the negative magnetophoretic force, a commercial water-based magnetite ferrofluid, EMG 408 (magnetic particle concentration 1.2% vol., nominal particle diameter 10 nm Ferrotec Co., Singapore) was used. The PEO solutions were then mixed with water-based ferrofluid (1:1) to obtain solutions with PEO concentrations of 250 ppm, 500 ppm, and 1000 ppm.

### Experimental setup

The sample solution was injected into the microfluidic device using 1 mL syringes and tygon tubing. Syringe pumps (PHD22/2000, Harvard Apparatus, USA) were used to maintain a constant flow rate. A non-uniform magnetic field was generated using two neodymium magnets placed in the microfluidic device. Particle trajectories were observed using an inverted fluorescence microscope (Olympus IX73, Japan). To gain comparable fluorescent data, the exposure time of the camera was fixed to 1 second.

### Microparticle and cell separation analysis

To facilitate optical microscope observation of the microparticle movement in the microfluidic channel, we used 10 µm polystyrene green fluorescent microparticles (1.05 g/mL density, Spherotech, USA) and 16 µm polystyrene red fluorescent microparticles (1.05 g/mL density, Spherotech, USA). For validation of the separation of the microfluidic channels, the green and red fluorescent polystyrene microparticle mixture was injected into the PEO mixed ferrofluid solution. In addition, 2–5 µm neural stem cells (NSCs) and 10–15 µm metastatic U87MG human glioblastoma cancer cells were used instead of the microparticles. CellTrace 5,6-carboxyfluorescein diacetate succinimidyl ester (CFSE) staining solution (Thermo Fisher Scientific Inc., NY, USA) was used for U87MG glioblastoma cancer cells and CellTrace Far red staining solution (Thermo Fisher Scientific Inc., NY, USA) was used for NSC staining. The cells were gently re-suspended and were subsequently incubated at 37 °C for 20 minutes. The staining solution was removed by the centrifuge and the suspended cells were then injected 1 × 10^6^ cells/mL suspension in medium into the microfluidic device. The fluorescent intensity was obtained from Image J software (National Institute of Health, USA) and the extracted data was analyzed using Matlab R2016a (Mathworks, USA).

## Results and Discussion

### Fabrication and numerical analysis of microfluidic separation device

**Figure 2:**
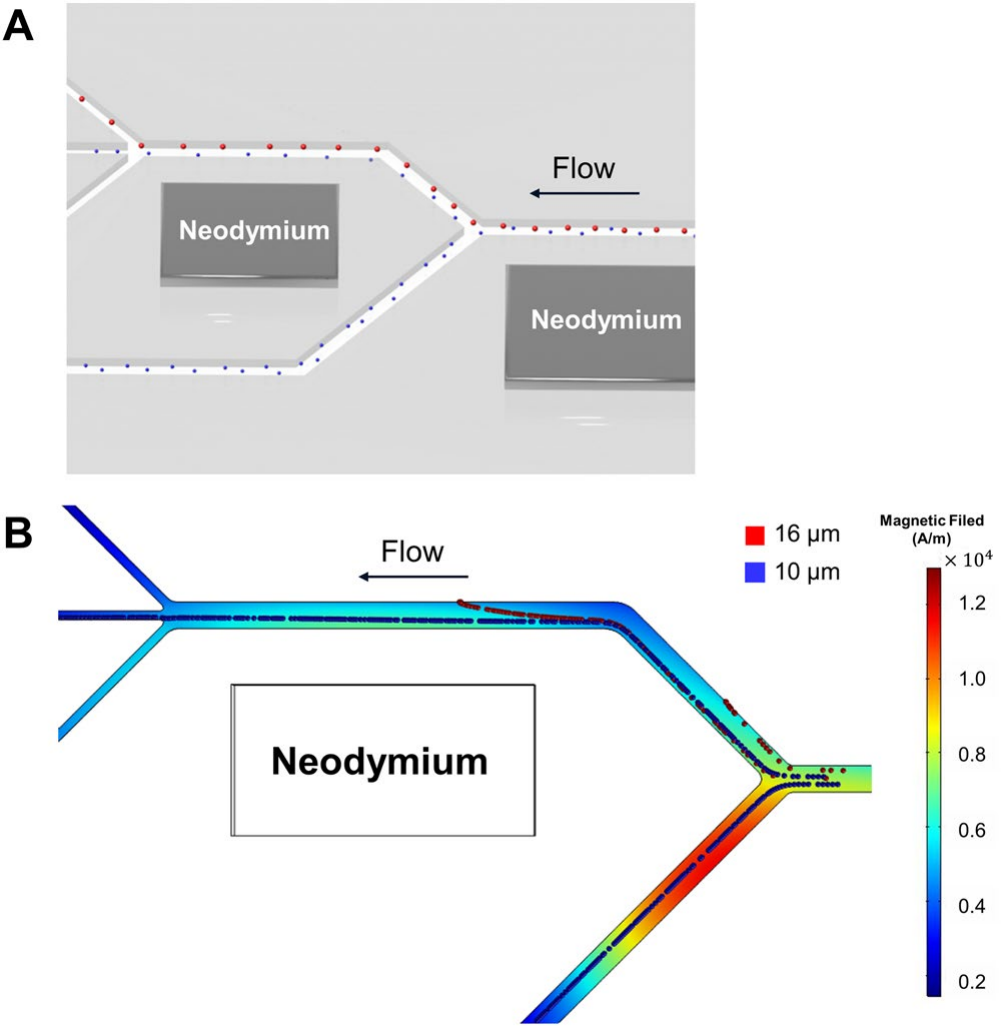
Computational simulation of the microfluidic separation device. (**A**) Schematic drawing of the position of the microparticles in the microfluidic separation device. (**B**) Simulation result of the microparticle tracing and magnetic field inside microfluidic separation devices. The red and green color represents 16 µm and 10 µm diameter microparticles, respectively.

The microfluidic separation device consisted of one inlet and four outlets, three outlets (Outlet 2~4) were used to separate the smaller microparticles, and one outlet (Outlet 1) was used to separate the larger microparticles (Fig. 1A). To prevent particle-particle interactions, the concentration of particles was decreased in a first sorting step, in which particles were sorted according to their size (Fig. 1C). Smaller-sized microparticles were partially discharged to the outlet located at the bottom (Outlet 4), decreasing the total microparticle density of the medium in the sorting region. In a second stage, microparticles were completely separated according to size in the separation region of the microfluidic device. To validate the design of our microfluidic separation device with dual-neodymium magnets, computational simulation using COMSOL Multiphysics 5.3 (COMSOL, Inc., USA) was also performed. The particles trajectories in magnetic field were simulated to show the negative magnetophoretic effect (Fig. 2). By introducing the magnetic field and negative magnetophoretic force, the microparticles were aligned toward the opposite side to the permanent magnet as previously described^49^. Aside from the negative magnetophoretic force, the inertial lift force and hydrodynamic drag force were also taken into consideration for the simulations. When the strength of magnets and the concentration of the ferrofluid used for the model were fixed to the same values as used in experiments, it was found that the best separation performance was at a sample flow rate of 7 µL/min. Further the model predicts that at lower flow rates (~5 µL/min) the separation performance decreases with the majority of particles being sorted towards Outlet 1. In contrast, at higher flow rates (~10 µL/min), most particles remain on the center line of the separation channel and exit the device through Outlet 2 and 3. Following these simulation results, it was decided to consider sample flow rates ranging from 3 to 10 µL/min in subsequent experiments. Furthermore, we expect an improvement to the sorting and separation efficiencies by the viscoelastic effect that was neglected in the simulations.

### Size-dependent separation of microparticles in the microfluidic device

**Figure 3:**
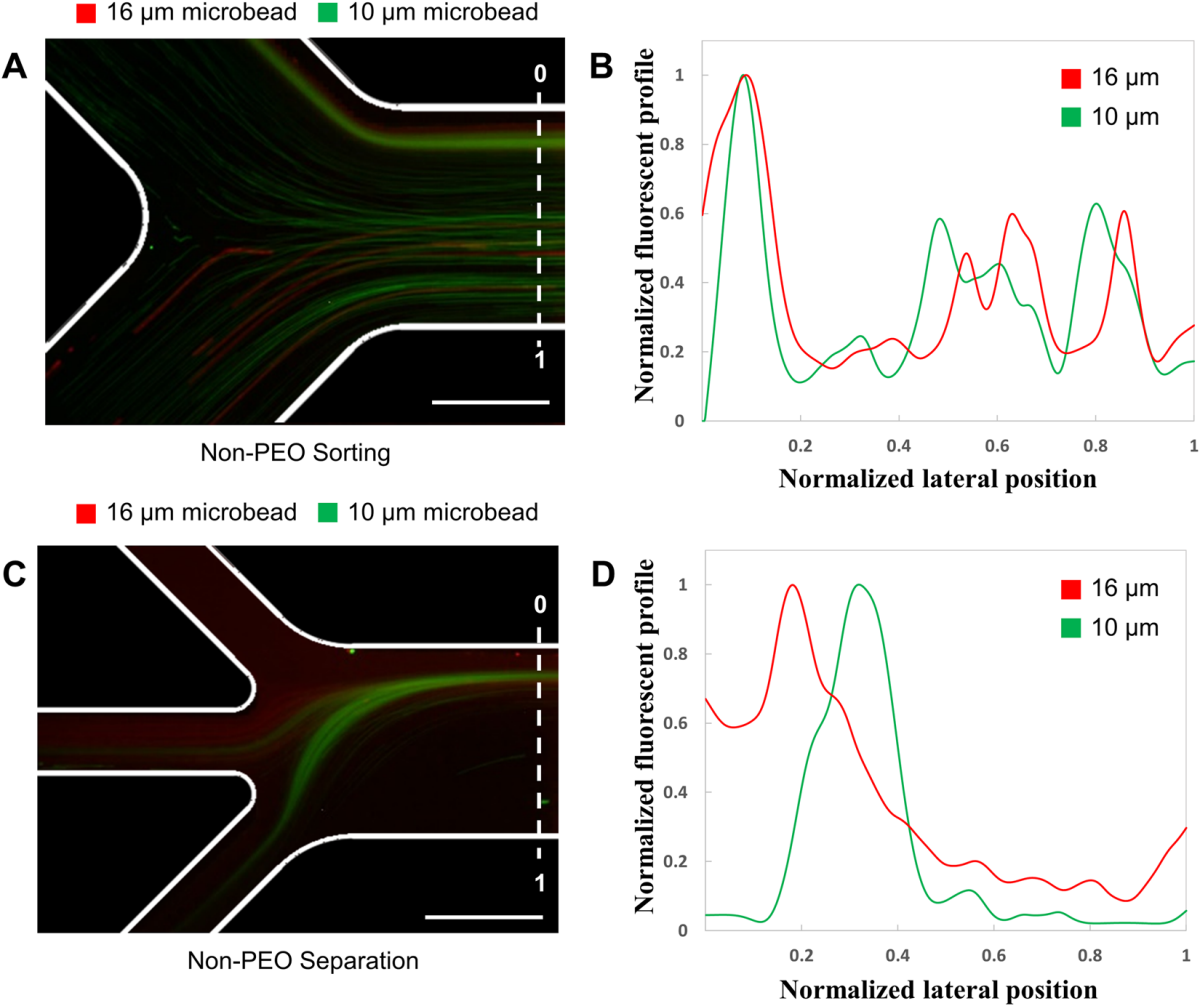
Validation of usage of PEO for viscoelastic flow. (**A**) Non-PEO experimental result with two types of fluorescent polystyrene microparticles with different sizes at the sorting region. Red color indicates 16 µm microparticles and green color indicates 10 µm microparticles. (**B**) Graph of the normalized fluorescent profile at the sorting region. The X-axis shows the normalized lateral position. (**C**) Non-PEO experimental result at the separation region. (**D**) Graph of normalized fluorescent profiles at the separation region. Scale bars are 500 µm.

To generate a viscoelastic force on the particles, PEO was added to the particle solution to obtain a non-Newtonian viscoelastic flow inside of the microfluidic sorting device. In a first experiment, the effect of the viscoelastic force was determined qualitatively by observing the sorting and separation of microparticles without the addition of PEO (Fig. 3). All other flow conditions were chosen in accordance with the previously conducted simulations. As can be seen from Fig. 3A, the particles were distributed randomly along the cross-section of the microchannel, leading to a loss of target particles though Outlet 4. This can also be seen when looking at the distribution of fluorescence intensity across the width of the microchannel at the end of the sorting region (Fig. 3B). A partial separation of the particles could then be observed in the separation region of the microfluidic device (Fig. 3C). However, as the particles entered the separation region without previous alignment from the sorting region, a mixture of the two microparticle sizes could be found in the center of the channel at the end of the separation region (Fig. 3D). This leads to a loss of the desired 16 μm particles through the middle outlet (Outlet 2). In the following it is demonstrated how the performance of the sorting and separation region can be increased by the addition of a viscoelastic force acting on the microparticles.

**Figure 4:**
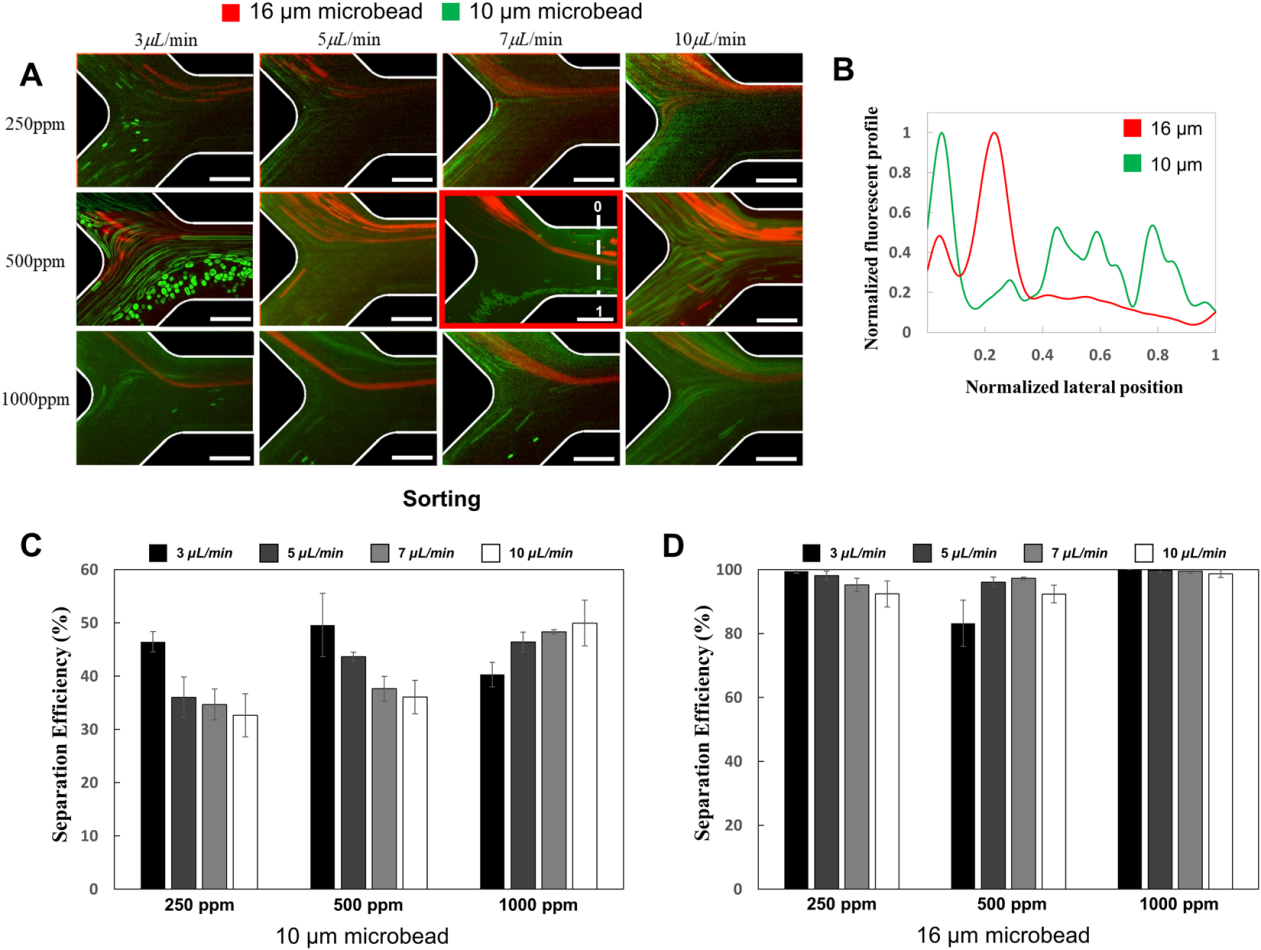
Microparticle behaviors at the sorting region in the microfluidic separation device. (**A**) Experimental result at the sorting region of the microfluidic device. The red rectangle represents the result at optimized condition. (**B**) Graph of the normalized fluorescent profile in the normalized lateral position at the sorting region for the optimized sorting conditions. Scale bars are 500 µm. Bar diagrams of sorting efficiencies for the smaller 10 µm diameter particles (**C**) and the larger 16 µm particles (**D**) for different sample flow rates and PEO concentrations.

**Figure 5:**
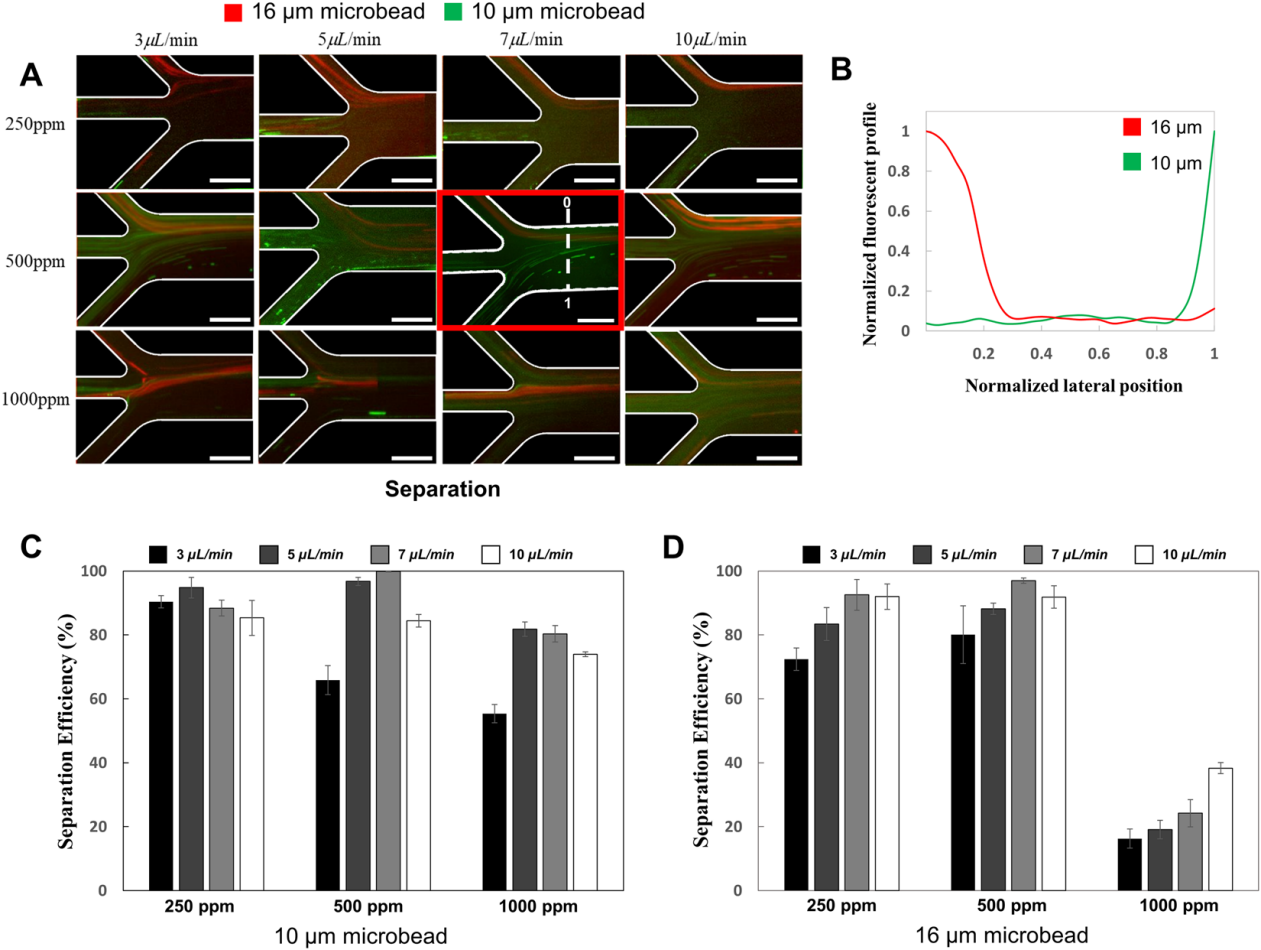
Microparticle behaviors at the separation region in the microfluidic device. (**A**) Experimental result at the separation region of the microfluidic separation device. The red rectangle represents the result at optimized condition. (**B**) Graph of normalized fluorescent profile in the normalized lateral position at the separation region for the optimized conditions. Scale bars are 500 µm. Bar diagrams of separation efficiencies for the smaller 10 µm diameter particles (**C**) and the larger 16 µm particles (**D**) for different sample flow rates and PEO concentrations.

In the following experiments, the PEO concentration and flow rate was varied to optimize the separation efficiency of the sorting and separation region (Figs 4, 5). At low concentrations of PEO (250 ppm), the negative magnetophoretic force is dominant (Π_*m*_ = 5.85 × 10^8^) and the particles become aligned along the wall of the sorting channel (Fig. 4A). In this case, the viscoelastic force on the particles is small (Wi = 0.023). When the flow rate was increased, the increase of the elastic force became more dominant than the increase of viscoelastic force, showing that the effect of the viscoelastic force on the particle trajectories could be negligible as previously described^50,51^. More than 90% of the 16 µm microparticles are sorted into the separation channel, while less than half of the 10 µm microparticles can be removed through Outlet 4 (Fig. 4C,D, Supplemental Fig. S1). The separation performance at 250 ppm concentration of PEO is dependent on the sample flow rate. At higher flow rates (7–10 µL/min), a separation efficiency of more than 90% is achieved at Outlet 1 for the 16 µm particles (Fig. 5D). For the smaller 10 µm particles, the separation efficiency is around 90% with no statistically relevant difference between the flow rates.

In contrast to the low PEO concentration, a concentration of 1000 ppm leads to an increase in the elasticity number (from 0.33 to 0.43), indicating a stronger effect of the viscoelastic forces compared to the inertial lift force. Through this, the particles could be aligned in the sorting region, leading to significant increases in sorting efficiencies for flow rates higher than 5 µL/min (Fig. 4C,D). In particular, the larger 16 µm diameter particles could be sorted towards the separation region with almost perfect efficiency (Supplemental Fig. S1F). In the separation region, however, a large decrease in separation efficiency was observed with less than 40% of the 16 µm diameter particles being collected from Outlet 1 (Fig. 5D). Simultaneously, the fraction of smaller 10 µm diameter microparticles collected from Outlet 1 were increased compared to the lower 250 ppm PEO experiments (Fig. 5C). This decrease in sorting efficiency can be attributed to the stronger viscoelastic force, aligning the particles in the center of the channel, countering the negative magnetophoretic force used for sorting.

As a high PEO concentration provided good sorting efficiencies and low separation efficiencies, and low PEO concentrations the opposite effect, further experiments were conducted with an intermediate concentration (500 ppm) of PEO. Through this, a balance between the negative magnetophoretic, viscoelastic, and inertial lift force could be achieved. The sorting efficiency of 97 ± 0.8% for the larger particles, and 38 ± 2.3% for the smaller particles at a flow rate of 7 µL/min could be achieved. The separation efficiency at the intermediate PEO concentration was also high with 97 ± 0.8% of the larger particles being recovered from outlet 1, and 99 ± 0.1% of the smaller particles recovered from Outlet 2, 3, and 4 (Supplemental Fig. S1). Furthermore, our experiments confirmed the results of the computer simulation, regarding the optimum flow rate to operate the microfluidic device. Therefore, we successfully demonstrated that 10 and 16 µm microparticles were sorted and separated from smaller particles with a high efficiency.

### Separation of brain cancer cells

**Figure 6:**
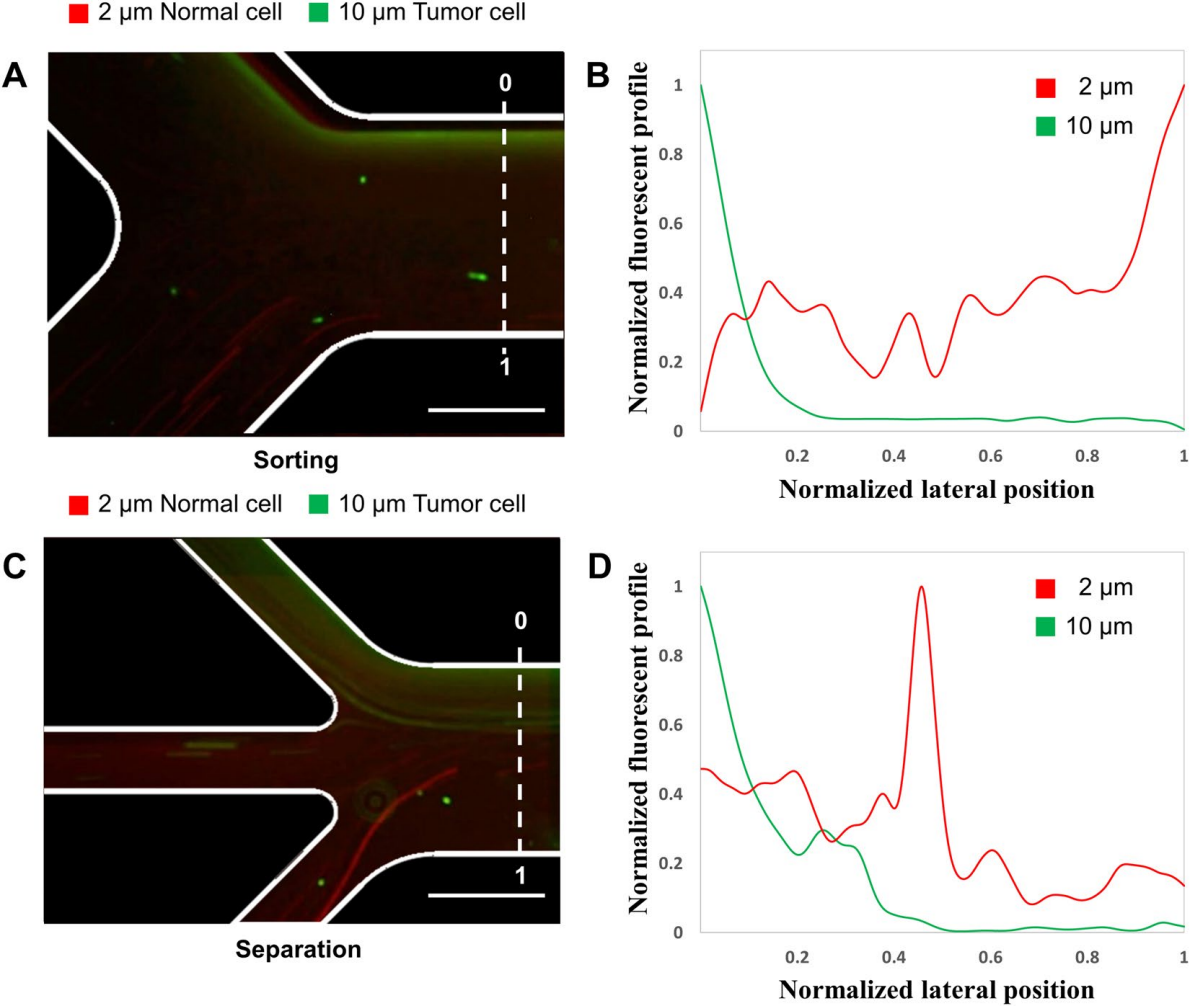
Brain cancer cell separation in the microfluidic device. (**A**) Experimental result with normal and cancer cells at the sorting region. Red color indicates 2 µm NSCs and green color indicates 10 µm glioblastoma cancer cells. (**B**) Graph of the normalized fluorescent profile along the channel cross-section at the end of the sorting region. The X-axis is shown as 0~1 in normalized lateral position at the sorting region. (**C**) Experimental result with normal and cancer cells at the separation region. (**D**) Graph of the normalized fluorescent along the channel cross-section at the end of the separation region. Scale bars are 500 µm.

To demonstrate the applicability of our microfluidic system for separation of cell samples, we further separated brain cancer cells in our microfluidic device. NSCs and glioblastoma cancer cells were injected into microfluidic separation devices with dual-neodymium magnets. The size of NSCs was approximately 2 μm and glioblastoma cancer cell was approximately 10 μm. Due to the difference in sample size compared to the microparticle experiments, the sample flow rate was adjusted to 13 µL/min. In the sorting region, the glioblastoma cancer cells were aligned on the opposite side of the magnet and NSCs were found at the wall on the magnet side (Fig. 6A). At the separation region, most glioblastoma cancer cells were observed at Outlet 1 and NSCs were observed at Outlet 2~4 (Fig. 6C). We confirmed that the microfluidic separation device with dual-neodymium magnets could separate two different cell types. Compared to microparticle separation experiments, the cell separation experiment was performed without any change of the microchannel design, PEO concentration, and ferrofluid concentration, except the flow rates. Applications for this separation could be used for cell therapy applications, where the glioblastoma cancer cells might be potentially treated by NSCs.

## Conclusions

We developed the microfluidic device with dual-neodymium magnets for size-dependent separation of microparticles and cells in a highly efficient manner using non-Newtonian viscoelastic PEO solution and ferrofluid. The viscoelasticity was used for focusing the microparticles to the center of the channel and the negative magnetophoresis was used for size-dependent separation of microparticles and cells. We confirmed that the position of the microparticles and cells in the microfluidic separation device with dual-neodymium magnets was regulated by flow rates and the concentrations of PEO. The best separation results could be achieved with a PEO concentration of 500 ppm and a flow rate of 7 μL/min for microparticles and 13 μL/min for the separation of brain cancer cells. Therefore, this microfluidic separation device with dual-neodymium magnets could be a potentially powerful tool to separate various small biomolecules in a controlled manner.

## Supplementary information

Supplementary Information
