# Supplementary material for: Dual-neodymium magnet-based microfluidic separation device
Source: Sci Rep. 2019 Jul 1;9:9502. doi: 10.1038/s41598-019-45929-y (PMC6602928; doi:10.1038/s41598-019-45929-y)
Supplement: Supplementary file 1 — Supplementary Information [file 41598_2019_45929_MOESM1_ESM.pdf]

Supplementary Information

Dual-neodymium magnet-based microfluidic separation device

Hyeon Gi Kye, Byeong Seon Park, Jong Min Lee, Min Gyu Song,
Han Gyeol Song, Christian D. Ahrberg, Bong Geun Chung*

Department of Mechanical Engineering, Sogang University, Seoul, Republic of Korea, 04107

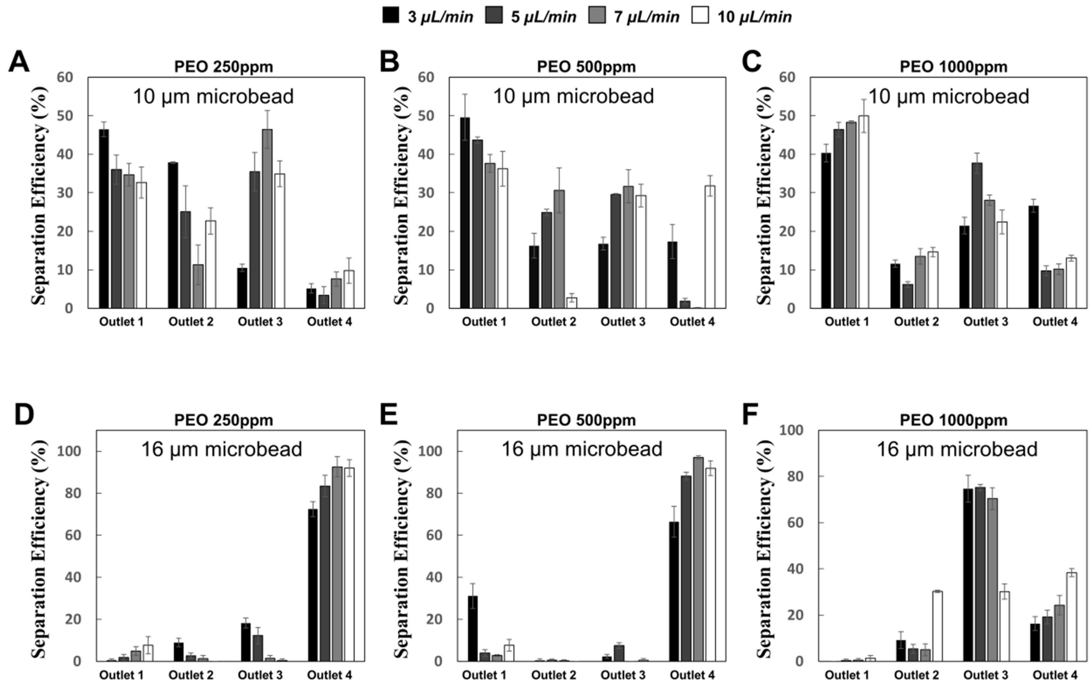

Supplemental Figure S1. Bar diagrams for the separation efficiencies of 10 µm particles for various flow rates at PEO concentrations of 250 ppm (A), 500 ppm (B), and 1000 ppm (C), indicating the fraction of particles calculated at each outlet. Bar diagrams for the separation efficiencies of 16 µm particles for various flow rates at PEO concentrations of 250 ppm (D), 500 ppm (E), and 1000 ppm (F), indicating the fraction of particles calculated at each outlet.
